# LRRC superfamily expression in stromal cells predicts the clinical prognosis and platinum resistance of ovarian cancer

**DOI:** 10.1186/s12920-023-01435-9

**Published:** 2023-01-18

**Authors:** Xiaoying Zhu, Shijing You, Xiuzhen Du, Kejuan Song, Teng Lv, Han Zhao, Qin Yao

**Affiliations:** 1 Department of Gynaecology, Affiliated Hospital of Qingdao University, Qingdao Medical College, Qingdao University, Qingdao, China; 2 Department of Pathology, Affiliated Hospital of Qingdao University, Qingdao Medical College, Qingdao University, Qingdao, China

**Keywords:** LRRC15, Ovarian cancer, Stroma, Primary platinum resistance, Immune microenvironment

## Abstract

**Background:**

Leucine-rich repeat sequence domains are known to mediate protein‒protein interactions. Recently, some studies showed that members of the leucine rich repeat containing (LRRC) protein superfamily may become new targets for the diagnosis and treatment of tumours. However, it is not known whether any of the LRRC superfamily genes is expressed in the stroma of ovarian cancer (OC) and is associated with prognosis.

**Methods:**

The clinical data and transcriptional profiles of OC patients from the public databases TCGA (n = 427), GTEx (n = 88) and GEO (GSE40266 and GSE40595) were analysed by R software. A nomogram model was also generated through R. An online public database was used for auxiliary analysis of prognosis, immune infiltration and protein‒protein interaction (PPI) networks. Immunohistochemistry and qPCR were performed to determine the protein and mRNA levels of genes in high-grade serous ovarian cancer (HGSC) tissues of participants and the MRC-5 cell line induced by TGF-β.

**Results:**

LRRC15 and LRRC32 were identified as differentially expressed genes from the LRRC superfamily by GEO transcriptome analysis. PPI network analysis suggested that they were most enriched in TGF-β signalling. The TCGA-GTEx analysis results showed that only LRRC15 was highly expressed in both cancer-associated fibroblasts (CAFs) and the tumour stroma of OC and was related to clinical prognosis. Based on this, we developed a nomogram model to predict the incidence of adverse outcomes in OC. Moreover, LRRC15 was positively correlated with CAF infiltration and negatively correlated with CD8 + T-cell infiltration. As a single indicator, LRRC15 had the highest accuracy (AUC = 0.920) in predicting the outcome of primary platinum resistance.

**Conclusions:**

The LRRC superfamily is related to the TGF-β pathway in the microenvironment of OC. LRRC15, as a stromal biomarker, can predict the clinical prognosis of HGSC and promote the immunosuppressive microenvironment. LRRC15 may be a potential therapeutic target for reversing primary resistance in OC.

**Supplementary Information:**

The online version contains supplementary material available at 10.1186/s12920-023-01435-9.

## Background

Ovarian cancer (OC) is a heterogeneous disease with a variety of subtypes [1]. With the emergence of high-throughput molecular techniques, high-grade serous cancers (HGSCs), the most common OC histologic subtype, were further stratified into 4 distinct molecular subtypes: mesenchymal, immune-reactive, proliferative, and differentiated [2]. A novel subtype of HGSC reflecting the mesenchymal cell type was also mentioned in the Tothill et al. [3] study, characterized by overexpression of N-cadherin and P-cadherin and low expression of differentiation markers, including CA125 and MUC1. However, little is known about how the interactions between cancer cells and the surrounding stromal microenvironment affect tumour growth and metastasis. Although many studies have been conducted on the biomarkers and treatment of human ovarian cancer [4–6], most biomarker studies and current treatment protocols for women with this disease are not subtype- or tumour-stromal specific. Given that the stroma has a supporting role in tumour progression and is characterized by a highly conserved proteomic signature in metastasis [7], we believe that looking for high expression and specific positioning in the OC stroma is particularly important.

Leucine rich repeat sequence (LRR) domains are known to mediate protein‒protein interactions [8]. Leucine rich repeat containing (LRRC) protein is widely distributed in the cytoplasm, cell membrane, cell nucleus and extracellular matrix, forming an evolutionarily conserved and multifunctional LRRC superfamily [9]. It has more than 4000 members, including cell adhesion molecules, RNA, enzyme inhibitors, tyrosine kinase, extracellular matrix and viral factors [10–13]. In recent years, there have been research reports that LRRC superfamily members have differences in their expression characteristics and roles in different types of malignant tumours [14–17], suggesting the complexity of LRRC superfamily members. However, it was not known whether any of the LRRC superfamily genes identified in any of these studies are also more highly expressed in HGSC stroma.

In contrast to those studies, our research is the first designed specifically to identify LRRC superfamily genes that are differentially expressed in HGSC stroma. We also included podoplanin (PDPN), which is highly and stably expressed in CAFs, [18, 19], to localize the gene expression origin in the stromal components as a reference. CAFs are unique, reprogrammed stromal cells with roles in cancer initiation, extracellular matrix remodelling, progression, premetastatic niche formation, and metastasis [20]. In a study on lung adenocarcinoma patients, PDPN + CAFs showed higher expression of TGFB1 and were associated with CD204 + TAM infiltration in stage I lung squamous cell cancer (SqCC), suggesting that PDPN + CAFs were associated with an immunosuppressive tumour microenvironment [21]. We aimed to determine whether the same result can be found in ovarian cancer. Tumour-infiltrating lymphocytes (TILs), as the main components of the immune microenvironment, include B cells, CD4 + T cells and CD8 + T cells [22, 23]. The expression of LRRC15 in the TIMER 2.0 database was positively correlated with CAF infiltration, which is consistent with our previous results. Interestingly, for the first time, we found a negative association between LRRC15 expression and CD8 + T-cell infiltration in OC. This suggested that LRRC15 + CAFs participate in the construction of an immunosuppressive microenvironment in ovarian cancer and promote immune escape. Immunotherapy for ovarian cancer is only effective for some people [24, 25]. Our results suggest that LRRC15 + CAFs in mesenchymal ovarian cancer may be used as a biological indicator to predict the proportion of patients who would likely respond to immunotherapy.

Platinum resistance is a difficult problem in the first-line treatment of ovarian cancer [26]; our results showed that it could also be predicted by the LRRC15 + CAF phenotype. LRRC15, PDPN, and CD8 have certain accuracy as single biomarkers in predicting the outcomes of platinum-based chemotherapy for ovarian cancer. LRRC15 is indeed related to the primary resistance of ovarian cancer, and treatments targeting LRRC15 may reverse ovarian cancer primary resistance.

## Methods

### Public databases analysis

The differentially expressed genes (DEGs) were downloaded as raw signals from Gene Expression Omnibus (http://www.ncbi.nlm.nih.gov/geo) under the accession numbers GSE40266 and GSE40595, interpreted, normalized and log2 scaled by R software (4.4.2) using the limma package. We included 86 samples for differential expression analysis. We downloaded the raw TCGA data from the GDC database (https://portal.gdc.cancer.gov/) and GTEx (http://www.gtexportal.org) database. GEPIA2 (http://gepia.cancer-pku.cn/) is an interactive web server developed by Peking University that is used to analyse cancer expression profile data. We analysed the overall survival (OS) of OC patients by using Kaplan‒Meier Plotter (http://kmplot.com/analysis/). We built PPI networks of LRRC15 on the GeneMANIA website (http://www.genemania.org). LinkedOmics (http://www.linkedomics.org) was used for Gene Ontology (GO) functional analysis and Kyoto Encyclopedia of Genes and Genomes (KEGG) pathway analysis. CAF and immune infiltration analyses were completed in TIMER2.0 (http://timer.cistrome.org/). The above public online database was established based on TCGA and GTEx. The UCSC Xena (https://xenabrowser.net/datapages/) database was used to obtain the RNA-seq data (TPM format) from TCGA and GTEx, which were uniformly processed by the Toil process.

### Nomogram model

We first divided LRRC15 into high expression and low expression groups according to the best cut-off value. The prognostic significance of LRRC15 expression and other clinicopathological variables was first assessed by univariate Cox regression analysis using overall survival time in a cohort of 371 HGSC patients from TCGA. Then, multivariate Cox regression analysis was performed to identify the prognostic effect of those factors. Finally, clinicopathological factors, including age, stage, and expression level of LRRC15, were integrated into a prognostic nomogram. The TCGA (n = 371) data were randomly divided into a training set and verification set at a ratio of 7:3. The models were also internally validated using calibration plots, and individually predicted 1-, 3-, and 5-year survival probabilities were generated to measure the predictive accuracy compared with the observed survival as “ground truth.” The predictive accuracies for overall survival were calculated using Harrell’s concordance index (C-index), which ranges from 0.5 (completely random prediction) to 1 (perfect prediction) [27]. A final nomogram was developed using the method with the greatest predictive accuracy for the individualized estimation of survival. All analyses were performed using R version 4.1.2. *P* values less than 0.05 were considered to be statistically significant.

### Cell culture and TGFβ-1 induction

The human foetal lung fibroblast cell line MRC-5 [28] was obtained from the cell bank of the Chinese Academy of Sciences. The cell lines were authenticated by their source organizations prior to purchase, routinely checked for mycoplasma contamination and used within 4 months after frozen aliquot recovery. DMEM/F-12 medium with 10% FBS and 1% penicillin/streptomycin (Thermo Scientific) was used to culture the cells. The cells were cultivated in culture flasks in a humidified incubator with 5% CO_2_ at 37 °C and 80% humidity. In the experiments, the cells were exposed to 5 µg/ml TGF-β1 for 48 h to transform into CAF phenotypes [29, 30].

### RNA extraction, reverse transcription PCR, and quantitative real-time polymerase chain reaction

TRIzol (Invitrogen, Carlsbad, CA, USA) was used to extract total RNA from cells. According to the protocol, total RNA was reverse transcribed into cDNA using two-step reverse transcription reagents (Promega, UK). SYBR Green Master (ROX) (Bimake.com) was used for q-PCR according to the manufacturer’s protocol. The relative gene expression of LRRC15 and PDPN was calculated by the 2^ − ΔΔCt^ method with the gene expression of GAPDH as a control. The primer sequences of LRRC15, PDPN and GAPDH are shown below (Table 1).

**Table 1:** Primer sequences

Primers	Sequences
LRRC15-forward	CCTGAGGATTGAGAAGAATGAGCTGTC
LRRC15-reverse	TTGTTGTTGGCGAGGCTGAGATAG
PDPN-forward	GTGCCGAAGATGATGTGGTGACTC
PDPN-reverse	GATGCGAATGCCTGTTACACTGTTG
GAPDH-forward	AGATCCCTCCAAAATCAAGTGG
GAPDH -reverse	GGCAGAGATGATGACCCTTTT

### Population and sample selection

From our patient database, we identified 1025 patients who underwent cytoreductive surgery for primary ovarian cancer at the Department of Gynaecology, Affiliated Hospital of Qingdao University, between January 1, 2014, and December 30, 2021. All patients underwent systematic surgical staging, and none of them received chemotherapy before surgery. Fresh frozen samples were reviewed by a pathologist who confirmed the presence of at least 70% tumour content. The histologic subtype and grade of the tumours were evaluated according to the World Health Organization criteria. Finally, we included 70 patients with primary HGSC. They were followed up to identify 10 cases of primary platinum resistance and further matched 20 platinum-sensitive cases at random from the patient database. Informed consent was obtained from all patients.

### Immunohistochemistry and pathology scoring

Immunohistochemistry (IHC) was performed on paraffin-embedded human HGSC tissues collected from the Department of Gynaecology, Affiliated Hospital of Qingdao University. The sections were deparaffinized in a xylene gradient and rehydrated in an ethanol gradient. Antigen retrieval was performed with a high pH buffer (DM828, Dako) at 97 °C for 20 min. Then, endogenous peroxidase was deactivated by applying 3% H_2_O_2_ in methanol. IHC of LRRC15, PDPN, and CD8 was performed by the Dako EnvisionTM method. Briefly, the Sects. (3 μm) were then incubated with anti-LRRC15 (NBP1-93,556, Novus), anti-PDPN (bs-1048R, Bioss) and anti-CD8 (bs-0648R, Bioss) antibodies. PBS buffer was used as negative control instead of primary antibody. This was followed by incubation with an anti-rabbit biotinylated secondary antibody (ab6721, Abcam) for 30 min. DAB peroxidase (ab64238, Abcam) was used as the final chromogen, and haematoxylin was used as the nuclear counterstain. Slides were scanned at × 10 magnification on a PANNORAMIC SCAN III instrument (3DHISTECH) at a resolution of 0.25 μm/pixel. The immunostaining scoring of LRRC15, PDPN, CD8 in each tissue was assessed using a scoring system encompassing staining intensity (0 = none, 1 = weak, 2 = moderate, and 3 = strong) and the proportion of expressing cells (0 = 0%, 1 = 1–25%, 2 = 26–50%, 3 = 51–75%, and 4 = 76–100%). The sum of the scores produced the final score, ranging from 0 to 12. Scores of 0 to 4 were defined as negative expression, and scores of 5 to 12 were defined as positive expression.

### Statistical analysis

Statistical details are available in the figure legends. Bioinformatic analysis, Cox regression analysis, nomogram model establishment and all statistical analyses were performed using R software (4.1.2). The real-time PCR data were analysed by the 2^−∆∆CT^ method. Associations between LRRC15, PDPN, and CD8 expression and the clinicopathological characteristics of the patients were analysed with the χ2 test (Fisher’s exact test). For normally distributed data, Student's *t test* was used to determine the statistical significance of differences. The Mann‒Whitney *U* test was used for nonparametric data. The data are presented as the mean ± standard deviation (SD). P < 0.05 was considered statistically significant.

## Results

### LRRC superfamily genes were overexpressed in HGSC stroma and CAFs

**Fig. 1:**
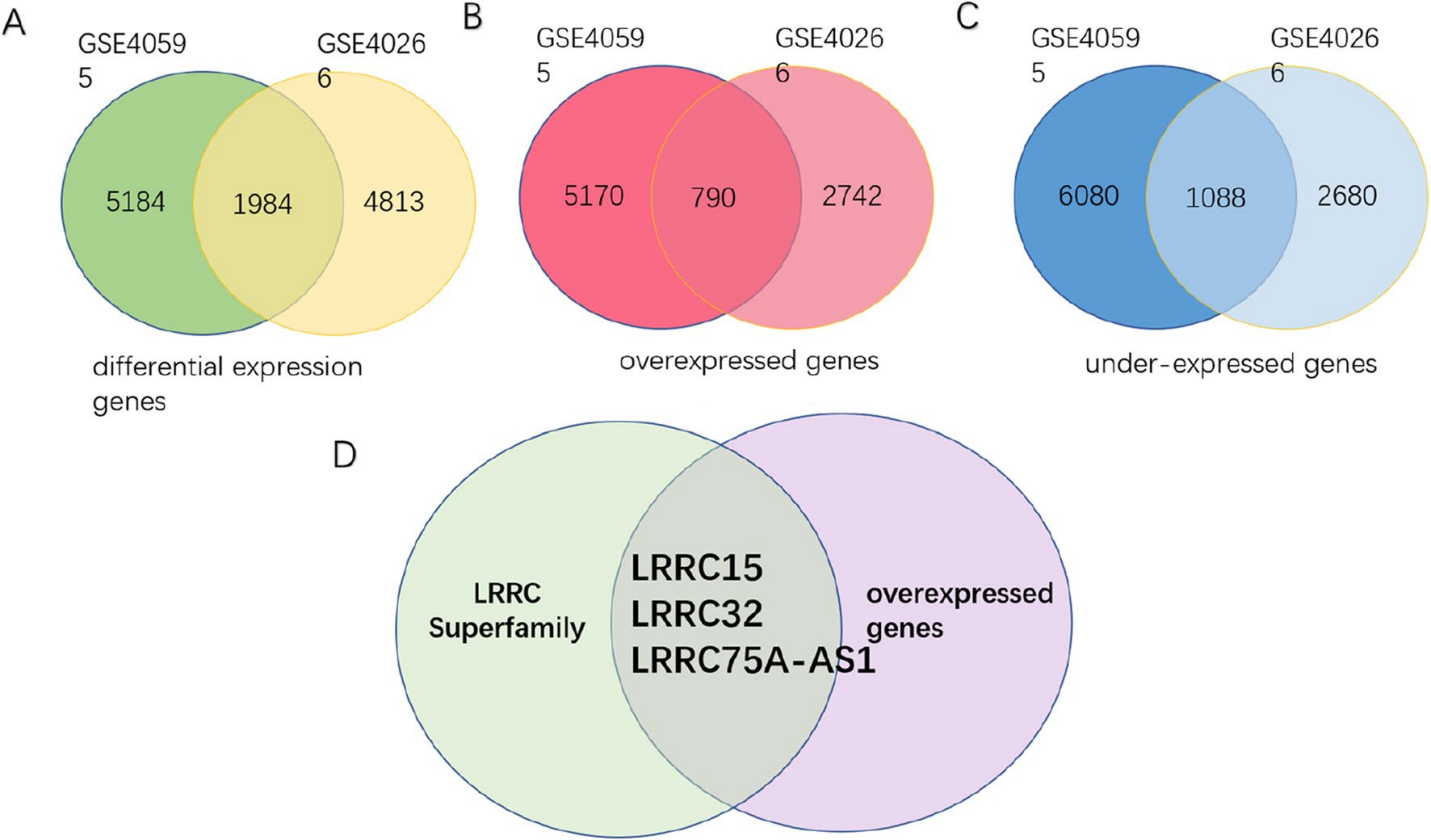
Venn diagram of differentially expressed genes. A total of 1,984 DEGs were expressed in both GSE40266 and GSE40595 (**A**). In total, 790 genes (**B**) were upregulated, and 1088 genes (**C**) were downregulated. The expression of LRRC15, LRRC32, and LRRC75A-AS1 from the LRRC superfamily was significantly increased in HGSC stroma and CAFs (*p* < 0.05) (**D**)

Two ovarian cancer stroma-related public datasets (GSE40266 and GSE40595) [31] were screened from the GEO database using the limma package in R software. Nine and 77 samples were included for differential expression analysis. In GSE40595, we conducted differential analysis of 31 tumour stroma samples and 6 normal ovarian stroma samples at the tissue level and obtained 5184 differentially expressed genes that met the established thresholds of |log_2_FC|> 1.5 and *p* < 0.05. As the most important cellular component in the tumour stroma, CAFs are thought to be formed by the transformation of TGF-β-treated NOF[30]. In GSE40266, 4813 gene signatures of the TGF-β response in ovarian fibroblasts were identified by transcriptional analysis of 6 TGF-beta-treated NOF151 cell line samples and 3 untreated NOF151 cell line samples. Then, the two groups of DEGs were compared, and a total of 1,984 overlapping DEGs (Fig. 1A) were obtained, including 790 upregulated genes (Fig. 1B) and 1088 downregulated genes observed in both datasets (Fig. 1C). We believe that these 790 genes are highly expressed in ovarian cancer stroma, especially in CAFs. Next, we identified LRRC15, LRRC32, and LRRC75A-AS1 as the three overexpressed gene signatures of the LRRC superfamily in ovarian cancer stroma (Fig. 1D). Among the three genes, LRRC75A-AS1 (also called small nucleolar RNA host gene 29, SNHG29) was excluded because it belongs to the lncRNA class. Therefore, LRRC15 and LRRC32 were found to be overexpressed in HGSC stroma and CAFs.

### LRRC15 is overexpressed in HGSC tumour tissues

**Fig. 2:**
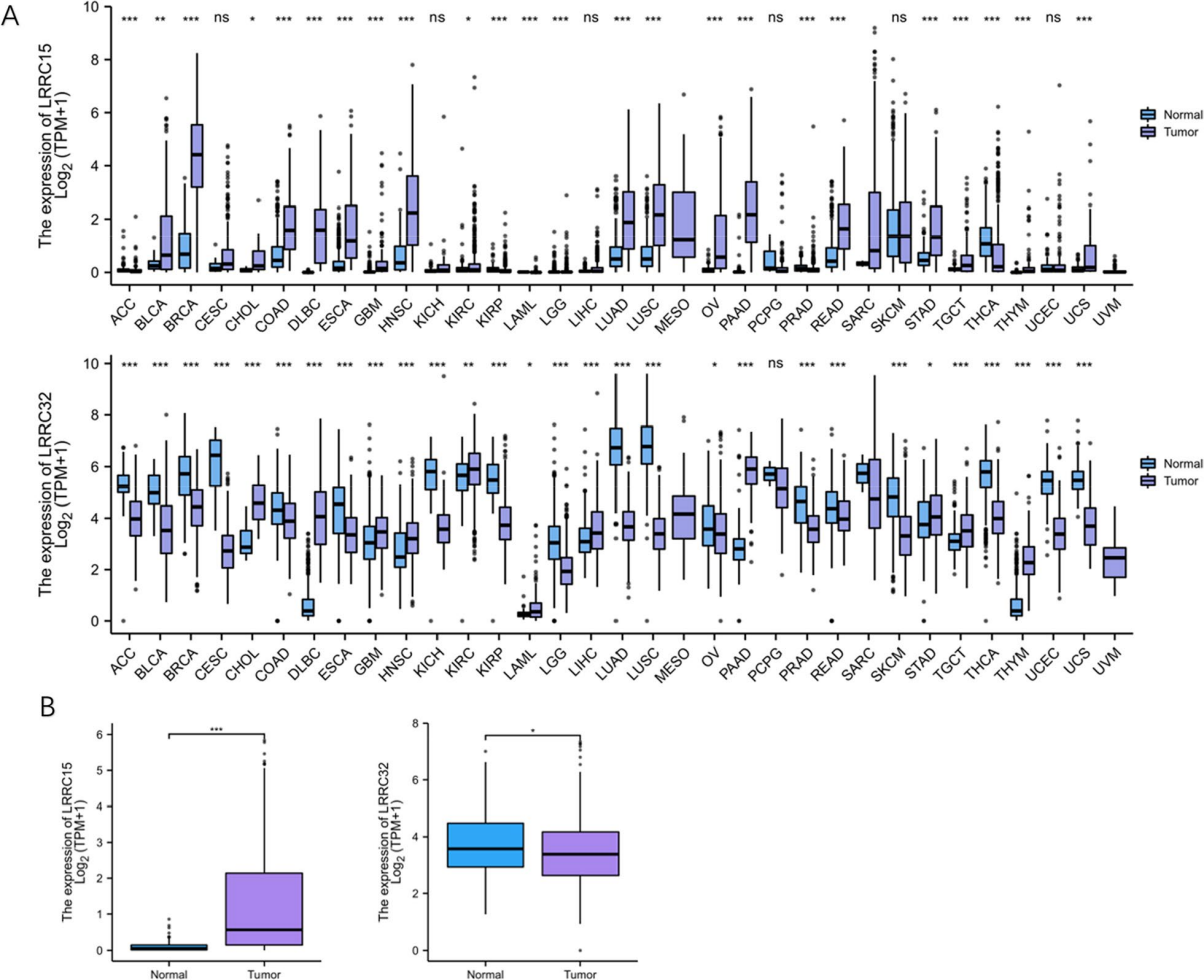
Box diagram for LRRC15 and LRRC32 expression. Pan-cancer analysis after log2 transformation using TPM-formatted RNA-seq data from TCGA and GTEx (A) (ns, *p ≥ 0.05; **p < 0.05; ***p < 0.01). The expression of LRRC15 in HGSC tumour tissues was significantly increased, *p* < 0.05. The expression of LRRC32 in HGSC tumour tissues was reduced, *p* > 0.05 (**B**)

Combined analysis of the mRNA expression profiles of pan-cancer samples and 515 HGSC samples (427 tumour + 88 normal) downloaded from UCSC Xena was performed. We used the limma R package to perform an intersample expression comparison of the data after log2 transformation. Pan-cancer analysis revealed that LRRC15 and LRRC32 are differentially expressed in multiple cancers (Fig. 2A). The expression differences of LRRC15 and LRRC32 in HGSC tumour tissues and normal ovarian epithelial tissues were visualized by the GGplot2 package in R software. We found that only LRRC15 was still highly expressed in tumour tissues (including stroma and parenchyma), and the differences were statistically significant (Figs. 2B). LRRC32 showed a downwards expression trend in OC tumour tissues overall (p > 0.05).

### TGF-β1-induced MRC-5 cells overexpress LRRC15

**Fig. 3:**
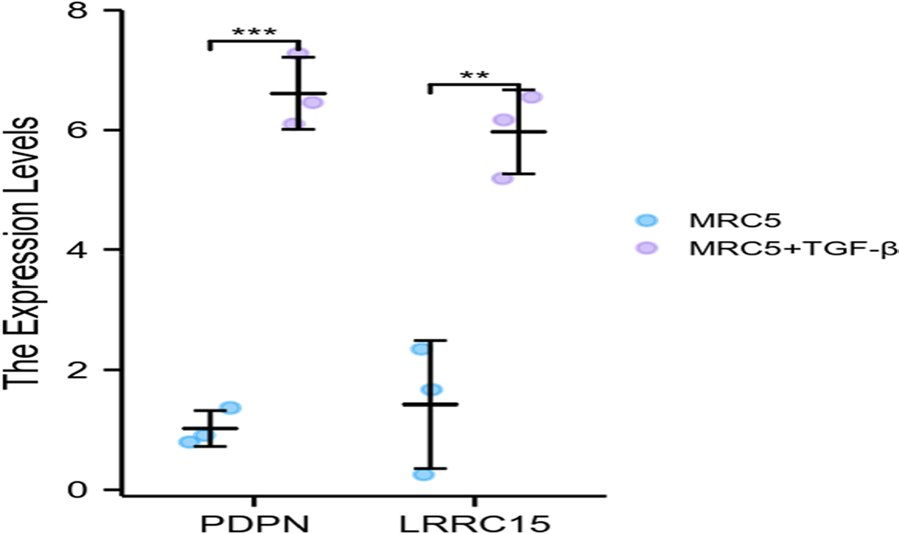
Scatter diagram for LRRC15 and PDPN. PDPN was overexpressed in the TGF-β1-treated MRC-5 cell line (*p* < 0.01). The expression of LRRC15 in the TGF-β1-treated MRC-5 cell line was also significantly increased (*p* < 0.05) (ns, *p ≥ 0.05; **p < 0.05; ***p < 0.01)

Transforming growth factor-β (TGF-β) is a pleiotropic factor that regulates cell differentiation and growth, tissue homeostasis and repair, and immune and inflammatory responses and plays an important role in tumour initiation and progression, functioning as both a suppressor and a promoter [32]. According to reports in recent years, the normal fibroblasts in many tumours are converted into CAFs under the action of TGF-β signalling, thereby promoting the growth and invasion of tumours [33–36]. In view of the results of the above public databases and datasets, the human embryonic lung fibroblast line MRC-5 was cultured in vitro and induced by TGF-β1 for 48 h to transform into a tumour stromal cell line with tumour-associated fibroblast characteristics [29, 37]. The literature showed that PDPN is a stably expressed gene in tumour-associated fibroblasts [38] and regulates the release of TGF-β through a positive feedback mechanism [39–42]. We sought to determine whether LRRC also has similar results and functions in stromal CAFs. As a reference, PDPN was also incorporated in the next study to evaluate the location and quantitative expression level of LRRC15. We quantitatively detected the mRNA expression levels of LRRC15 by three separate repeatable qPCR (Table 2). As expected, we found that LRRC15 was consistent with PDPN and showed higher expression in the MRC-5 cell line induced by TGF-β1 (*p* < 0.05) (Fig. 3A) (Additional file 1: Table S1). These results indicated that LRRC15 is indeed highly expressed in CAFs and may be involved in the regulation of the TGF-β pathway together with PDPN.

**Table 2:** mRNA expression of LRRC15 and PDPN in TGF-beta-treated and untreated MRC-5 cells

Group1	Group2	Number	Min	Max	Median	IQR	Lower quartile	Upper quartile	Mean	SD	SE
PDPN	MRC5	3	0.800	1.368	0.913	0.284	0.857	1.141	1.027	0.301	0.174
PDPN	MRC5 + TGF-β	3	6.100	7.271	6.463	0.586	6.282	6.867	6.612	0.599	0.346
LRRC15	MRC5	3	0.255	2.349	1.669	1.047	0.962	2.009	1.424	1.068	0.617
LRRC15	MRC5 + TGF-β	3	5.189	6.553	6.171	0.682	5.680	6.362	5.971	0.704	0.406

Statistical description of the mRNA expression of LRRC15 and PDPN in TGF-beta-treated and untreated MRC-5 cells: In the PDPN group, the mean levels were 1.027 ± 0.301 in the MRC5 group and 6.612 ± 0.599 in the MRC5 + TGF-β group. In the LRRC15 group, the mean levels were 1.424 ± 1.068 in the MRC5 group and 5.971.0.704 in the MRC5 + TGF-β group

### LRRC15 is associated with poor prognosis in OC

**Fig. 4:**
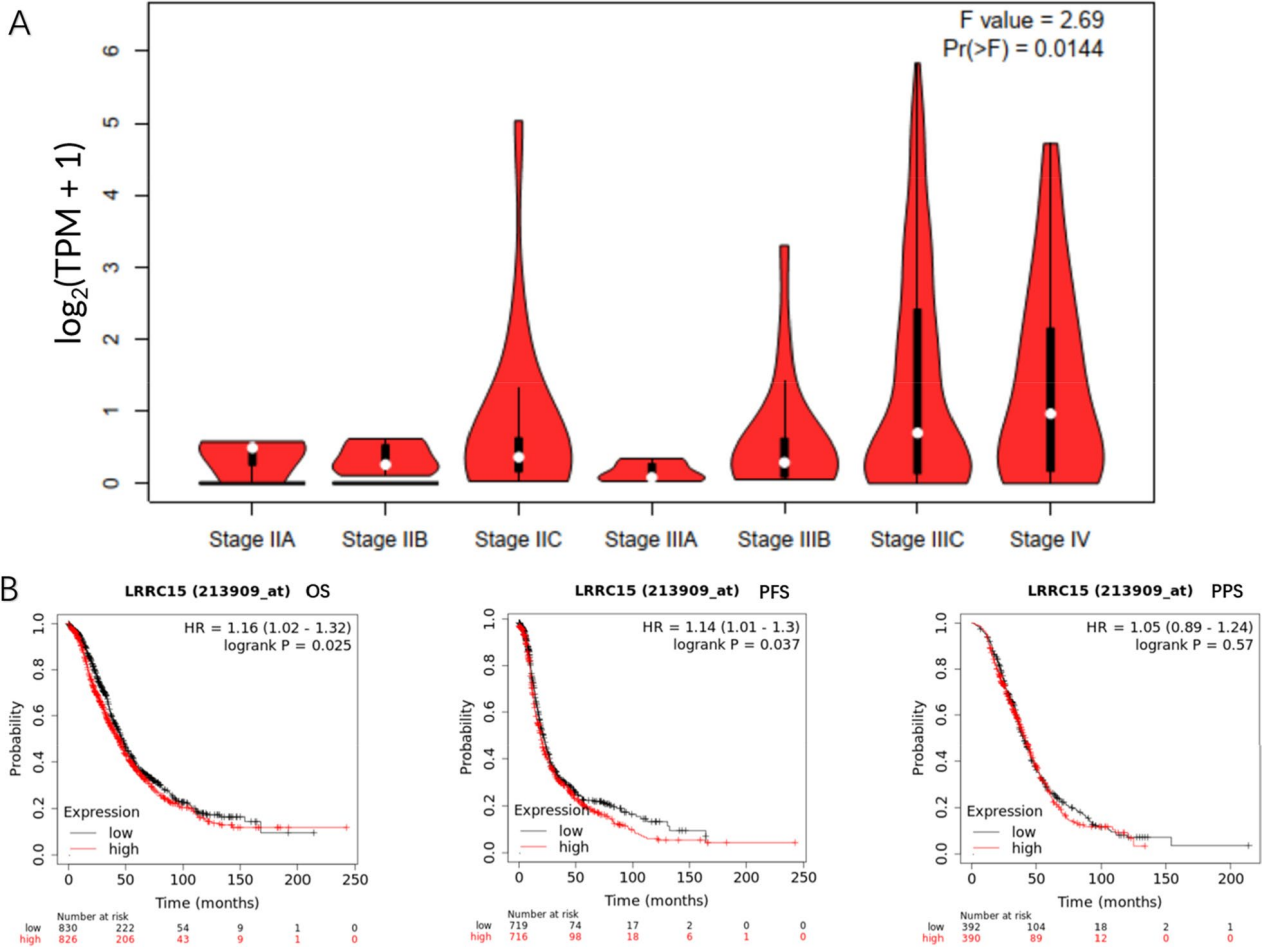
Prognostic analysis for LRRC15. High expression of LRRC15 was associated with later substages of HGSC in GEPIA2, *p* < 0.05 (**A**). LRRC15 expression was negatively correlated with OS, PFS and PPS of OC, *p* < 0.05. The Kaplan–Meier Plotter database parameter settings: LRRC15 probe was 213909_at, split patients by auto select best cut-off (OS: n = 1656, cut-off value used in analysis = 481; PFS: n = 1435, cut-off value used in analysis = 435; PPS: n = 782, cut-off value used in analysis = 444), others were default parameters (**B**)

We used R to obtain RNA-seq data from TCGA-OV (FPKM format converted to TPM format and log2 transformed) as well as relevant clinical information, which is regularly updated. The data were grouped by the median expression of LRRC15 (n = 379, median = 194). Further statistical analysis showed that the expression of LRRC15 was correlated with tumour stage and primary treatment outcome. High LRRC15 expression consistently predicted a later stage and worse primary treatment outcome (*p* < 0.05) (Table 3). Furthermore, the high expression of LRRC15, which is related to the substaging of OC, was confirmed in GEPIA2 (Fig. 4A). Kaplan–Meier Plotter (sources from the GEO, EGA, and TCGA databases) also showed that LRRC15 was significantly correlated with poor overall survival (OS) (n = 1656, HR = 1.16, *p* = 0.025), progression-free survival (PFS) (n = 1435, HR = 1.14, *p* = 0.037) in OC. The effect of Post progression survival (PPS) (n = 782, HR = 1.05, *p* = 0.57) on prognosis in the current sample size did not show statistical significance (Fig. 4B). The above are grouped by median. These results suggested that LRRC15 is associated with poor prognosis and might coordinate OC progression.

**Table 3:** Clinical data

Characteristic	Low expression of LRRC15	High expression of LRRC15	*p*	Statistic	Method
n	189	190			
*FIGO stage, n (%)*			0.008		Fisher.test
Stage I	0 (0%)	1 (0.3%)			
Stage II	18 (4.8%)	5 (1.3%)			
Stage III	147 (39.1%)	148 (39.4%)			
Stage IV	23 (6.1%)	34 (9%)			
*Primary therapy outcome, n (%)*			0.028	9.08	Chisq.test
PD	10 (3.2%)	17 (5.5%)			
SD	12 (3.9%)	10 (3.2%)			
PR	14 (4.5%)	29 (9.4%)			
CR	118 (38.3%)	98 (31.8%)			
*Race, n (%)*			0.732	0.62	Chisq.test
Asian	7 (1.9%)	5 (1.4%)			
Black or African American	14 (3.8%)	11 (3%)			
White	164 (44.9%)	164 (44.9%)			
*Age, n (%)*			0.963		
< = 60	103 (27.2%)	105 (27.7%)			
> 60	86 (22.7%)	85 (22.4%)			
*OS event, n (%)*			0.967		
Alive	74 (19.5%)	73 (19.3%)			
Dead	115 (30.3%)	117 (30.9%)			
*Histologic grade, n (%)*			0.430		
G1	0 (0%)	1 (0.3%)			
G2	20 (5.4%)	25 (6.8%)			
G3	164 (44.4%)	158 (42.8%)			
G4	1 (0.3%)	0 (0%)			
Age, median (IQR)	59 (51, 69)	59 (51, 67)	0.744	18,304	Wilcoxon

FIGO stage and histologic grade did not meet the condition of a theoretical frequency > 5 or a total sample quantity > 40, so Fisher's exact test was used. Each level of the primary therapy outcome, race, and OS met the conditions of a theoretical frequency> 5 and a total sample quantity> 40, and the chi-square test was used. Age did not satisfy a normal distribution (P <0.05), and the Wilcoxon rank sum test was used.

### Independent prognostic analysis of LRRC15 and development of a nomogram

**Fig. 5:**
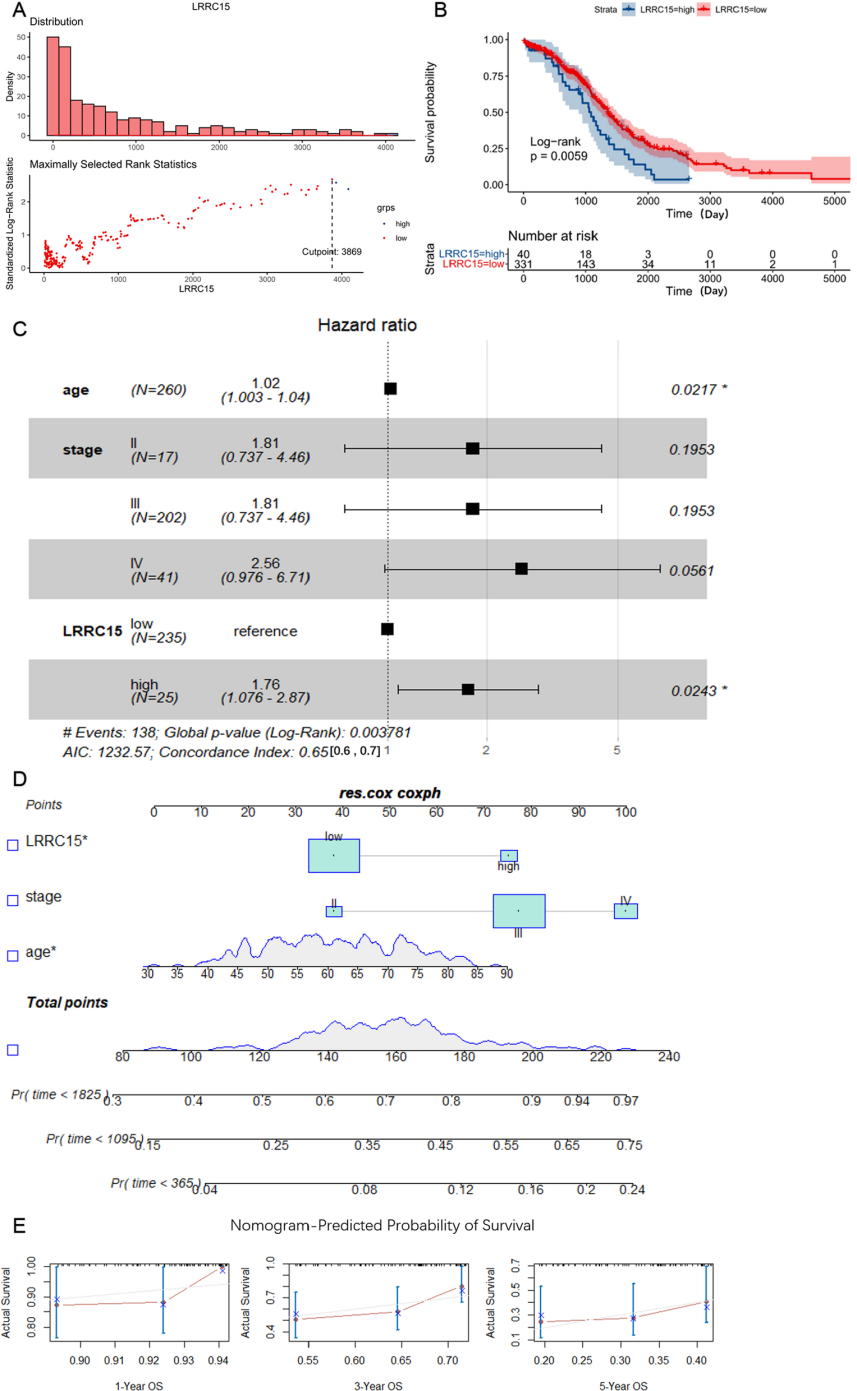
Construction and validation of a nomogram in TCGA. The best cut-off value of LRRC15 (**A**). The LRRC15 expression level was divided into high and low groups with the best cut-off value. The K-M curve shows that the LRRC15-high group had a shorter OS, P < 0.05 (**B**). Multivariate Cox regression forest plot based on the TCGA training set (n = 260). C-index = 0.65 (0.6, 0.7) (**C**). The nomogram based on LRRC15 expression levels and clinicopathological factors, including age and FIGO stage (**D**). Calibration curves for the nomogram predicting 1-, 3-, and 5-year survival in OV patients from the TCGA verification set (n = 111) (**E**)

Next, we further analysed the above data and found that the best cut-off value of LRRC15 was 3689 through the R survivalROC package (Fig. 5A). Then, the patients were redivided into the LRRC15-high group and LRRC15-low group according to the best cut-off value. Again, K-M analysis showed that the LRRC15-high group had a shorter OS (Fig. 5B). This is consistent with the findings of previous research. We randomly divided the TCGA-OV data (n = 371) into a training set (n = 260) and verification set (n = 111) at a ratio of 7:3. We next conducted univariate and multivariate Cox regression analyses to evaluate the influence of clinicopathological factors and LRRC15 expression on the OS of the training set (Table 4). As shown in Table 4, age and LRRC15 were independent prognostic factors in both analyses. We further visualized the results in a forest plot (Fig. 5C). Moreover, we established a prognostic nomogram in the TCGA-OV training set that included LRRC15 expression levels and clinicopathological factors, including age and stage (Fig. 5D). The 1-, 3-, and 5-year OS calibration curves (Fig. 5E) in the verification set were close to the ideal curve, showing that the nomogram could accurately predict the prognosis of patients with OC.

**Table 4:** Univariable and multivariable analyses of LRRC15 and clinical factors in TCGA

Variables	Univariable analysis				Multivariable analysis			
	HR	95% CI of HR		*p*	HR	95% CI of HR		*p*
		Lower	Upper			Lower	Upper	
Age	1.021	1.004	1.038	0.0132*	1.019*	1.0028	1..036	0.0217
Stage III	1.992	0.8115	4.888	0.1326	1.813	0.7367	4.463	0.1953
Stage IV	2.798	1.0707	7.312	0.0358*	2.559	0.9759	6.710	0.0561
LRRC15-high	1.874	1.164	3.019	0.00977**	1.757*	1.0758	2.871	0.0243

Significance: ns; *p* < 0.05; *p < 0.01; **p < 0.001

### Gene set enrichment analysis of the LRRC superfamily in ovarian cancer

**Fig. 6:**
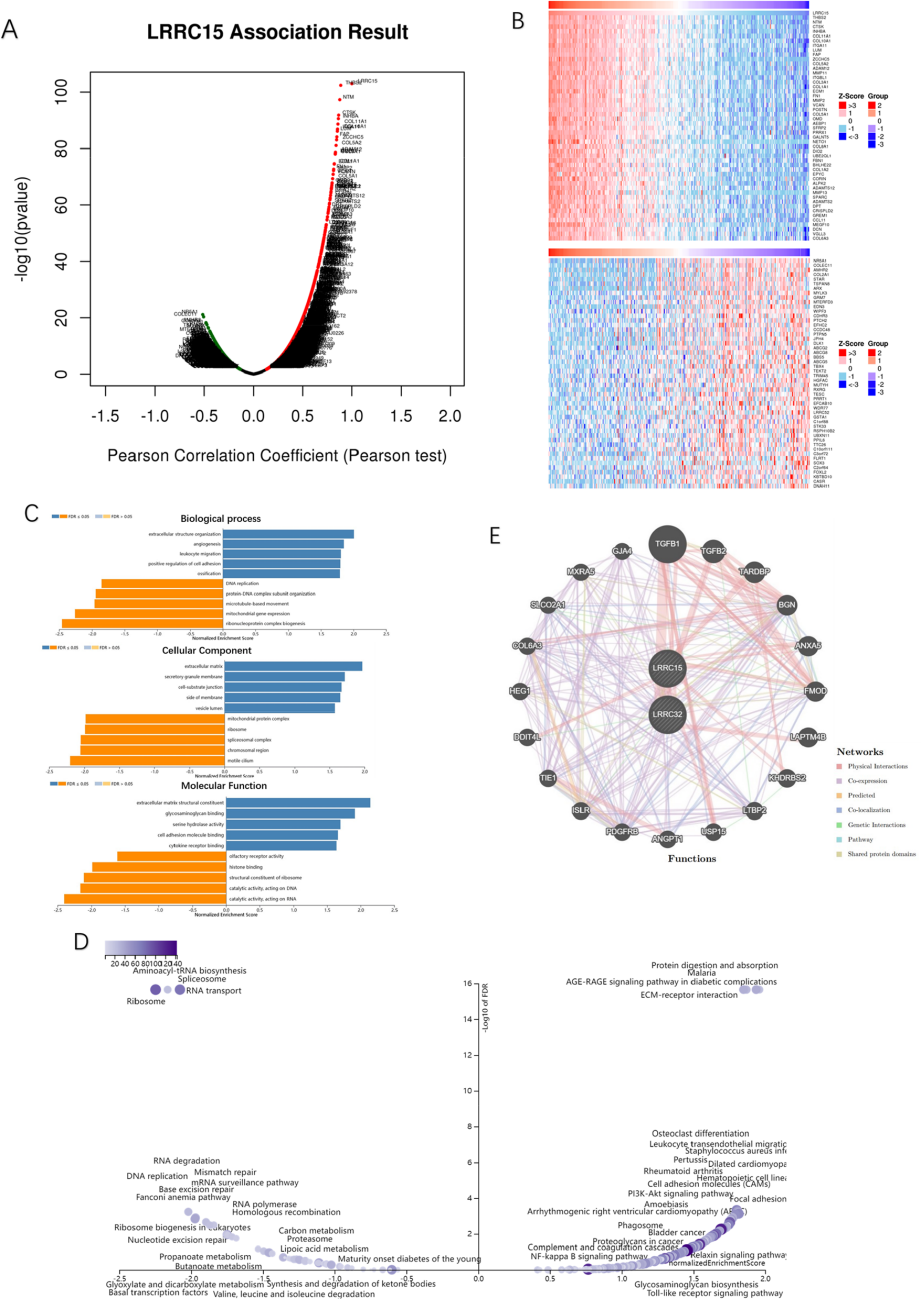
GSEA for LRRC15. LRRC15 coexpressed genes in OC (LinkedOmics). The global LRRC15 highly correlated genes identified by Pearson’s test in the OC cohort (**A**). Heatmaps showing the top 50 genes positively and negatively correlated with LRRC15 in OC (**B**). Red indicates positively correlated genes, and blue indicates negatively correlated genes. Significantly enriched GO annotations (**C**) and KEGG pathways (**D**) of LRRC15 in the OC cohort. PPI network analysis was performed in GeneMANIA for the two gene signatures identified in GSE40266 and GSE40595. The results showed that LRR15 and LRRC32 were significantly correlated with the TGF-β pathway

On the website LinkedOmics, we analysed the genes most related to LRRC15 using data from 581 ovarian cancer patients from the TCGA database. Among the 6426 genes, 3306 genes (*p* < 0.05) and 2506 genes (*p* < 0.01) were significantly correlated. As shown in the association curve, 1567 genes (red dots) showed a significantly positive correlation with LRRC15, whereas 1739 genes (green dots) showed a significantly negative correlation with LRRC15 (*p* < 0.05) (Fig. 6A). The top 50 genes of both are shown in the heatmap (Fig. 6B). We found that the 10 most relevant genes were THBS2, NTM, CTSK, INHBA, COL11A1, COL10A1, ITGA11, LUM, FAP, and ZCCHC5. Gene set enrichment analysis (GSEA) was further carried out. Analysis of significiant GO terms while maximizing gene coverage showed that the top genes significantly positively correlated with LRRC15 primarily participated in extracellular structure organization, angiogenesis, leukocyte migration, positive regulation of cell adhesion, ossification, etc. They are mainly expressed on extracellular matrix components and could perform the molecular functions of extracellular matrix structural constituent, glycosaminoglycan binding, serine hydrolase activity, cell adhesion molecule binding, and cytokine receptor binding (Figs. 6C). In the KEGG pathway analysis[43–45] of these genes, we found that they were significantly enriched in the following pathways: protein digestion and absorption, ECM-receptor interaction, PI3K-Akt signalling pathway, etc. (Figs. 6D). We next explored which pathways related to HGSC progression the LRRC superfamily play a role in. We performed PPI network analysis in GeneMANIA for the previously identified LRRC15 and LRRC32 genes, which are highly expressed in the stroma of HGSC (Figs. 6E). The results showed that they were the richest in the TGF-β protein family. This again demonstrates that the LRRC superfamily may be involved in the tumour-promoting effects of TGF-β in the progression of HGSC, such as immunosuppression, angiogenesis, metastasis, epithelial–mesenchymal transformation (EMT) of tumour cells, and fibroblast activation.

### LRRC15 affects the infiltration of CD8 + T cells and CAFs

**Fig. 7:**
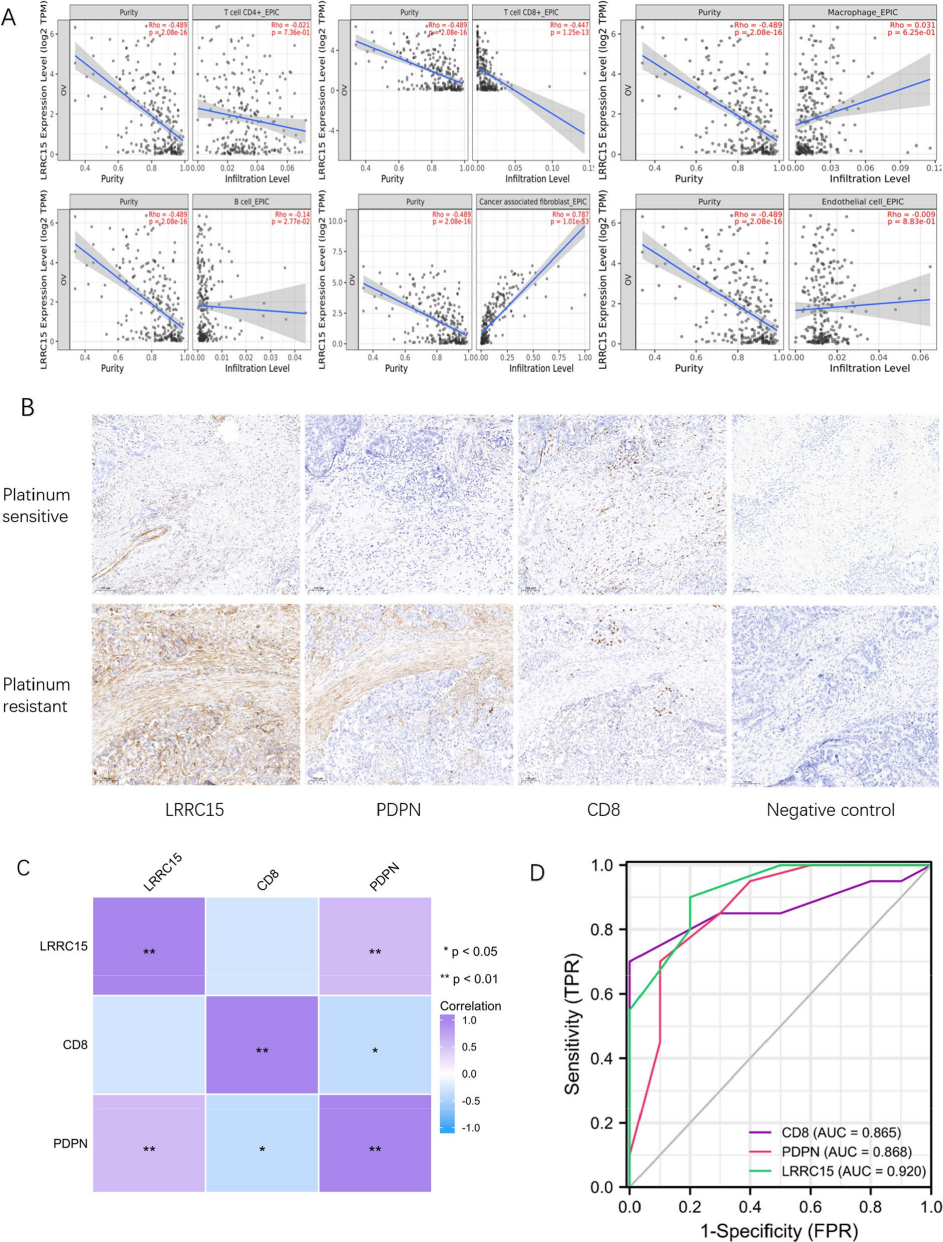
Relationship between LRRC15 expression and infiltration of immune cells. The EPIC method was used in TIMER2.0 for correlation analysis, and the results showed statistical significance **A** IHC slides were scanned at × 10 magnification on the PANNORAMIC SCAN III instrument (3DHISTECH) at a resolution of 0.25 μm/pixel. The figure shows the expression of LRRC15, PDPN and CD8 in platinum-resistant and platinum-sensitive patients **B** Spearman correlation analysis of LRRC115, PDPN and CD8. LRRC15 and PDPN were positively correlated (r = 0.56, *p* < 0.01). LRRC15 and CD8 were negatively correlated (r = −0.28, *p* = 0.13). CD8 and PDPN were negatively correlated (r = −0.38, *p* = 0.04) **C** The diagnostic ROC curve for LRRC15, PDPN and CD8 is an independent indicator **D** CD8 has certain accuracy (AUC = 0.865, CI = 0.736–0.994), PDPN has certain accuracy (AUC = 0.868, CI = 0.716–1.000), and LRRC15 has high accuracy (AUC = 0.920, CI = 0.825–1.000) in predicting drug resistance and sensitive outcomes. *The area under the ROC curve is between 0.5 and 1. The closer the AUC is to 1, the better the diagnostic effect. The lower the accuracy is when the AUC is 0.5–0.7, the certain the accuracy is when the AUC is 0.7–0.9, and the higher the accuracy is when the AUC is above 0.9

Since we found a positive correlation between LRRC15 and the tumour immunosuppressive molecule TGF-β in PPI networks, we speculated that LRRC15 might be involved in the OC immune infiltration or exclusion phenotype proposed by Hegde et al.[46]. Therefore, we analysed the relationship between LRRC15 and the infiltration of various immune cells in the TIMER2.0 database using EPIC (Estimating the Proportions of Immune and Cancer cells). First, we observed a negative correlation between LRRC15 and tumour purity. That is, the high expression of LRRC15 is not mainly reflected in the tumour parenchyma but may promote the proliferation of HGSC stroma. The major results showed that the high expression of LRRC15 was negatively correlated with CD8 + T-cell infiltration but positively correlated with CAFs (*p* < 0.05) (Fig. 7A). Next, we further confirmed this result by IHC staining of surface markers of T cells and CAFs. In the 30 HGSC patients we ultimately included, we used a scoring system encompassing the staining intensity (0 = none, 1 = weak, 2 = moderate, and 3 = strong) and the proportion of expressing cells (0 = 0%, 1 = 1–25%, 2 = 26–50%, 3 = 51–75%, and 4 = 76–100%). The positive expression rates of LRRC15, PDPN and CD8 were 63.3%, 50.0% and 66.7%, respectively (Table 5). We found that LRRC15 is always expressed at the same location as PDPN, and LRRC15 has a wider range of positive expression in stromal components in addition to CAFs, such as basal cells, endothelial cells, and Langerhans cells. Meanwhile, we observed that CD8 + T-cell infiltration decreased with increasing LRRC15 expression in the tissues of patients with advanced ovarian cancer (Fig. 7B). Then, we used the R ggplot2 package to calculate the Spearman correlation for the final score. The results showed that the expression of LRRC15 was positively correlated with that of PDPN (*p* < 0.01) and negatively correlated with that of CD8 (*p* > 0.05). However, there was a significant positive correlation between the expression of PDPN and CD8 (*p* < 0.05) (Fig. 7C). In summary, the expression of LRRC15 contributes to the proliferation of PDPN + CAFs and promotes the formation of mesenchymal ovarian cancer. To a certain extent, the infiltration of CD8 + T cells was inhibited, and the formation of an immunosuppressive microenvironment was promoted. These results still need to be verified with a larger sample size, which is the aim of our follow-up work.

**Table 5:** IHC results for LRRC15, PDPN and CD8

Gene	Number of samples	
	Positive	Negative
*n = 30*		
LRRC15	19 (63.3%)	11 (36.7%)
PDPN	15 (50.0%)	15 (50.0%)
CD8	20 (66.7%)	10 (33.3%)

### LRRC15 + HGSC is related to primary platinum resistance

Although platinum-based chemotherapy has always been the first-line treatment for ovarian cancer, primary platinum resistance has become a bottleneck in ovarian cancer treatment worldwide. Therefore, we screened 10 patients with clinically diagnosed primary platinum resistance from our cases and randomly matched 20 patients with platinum sensitivity. All patients received complete initial platinum-containing chemotherapy after the operation. A platinum-free interval < 6 months was defined as platinum resistance, and that > 6 months was defined as platinum sensitivity. We used R software on the clinical data and IHC results for further statistical analysis (Table 6). The results showed that the expression scores of LRRC15 and PDPN were higher in platinum-resistant patients, and the CD8 expression scores were lower (*p* < 0.01), but the reverse was true in platinum-sensitive patients. A diagnostic ROC (receiver operating characteristic) curve was used to determine the diagnostic significance of LRRC15, PDPN and CD8 as three independent indicators for primary platinum-resistant ovarian cancer. We observed that the predictive ability of LRRC15 was relatively accurate (AUC = 0.920, CI = 0.825–1.000) in predicting drug resistance and sensitivity outcomes (Fig. 7D). This indicates that LRRC15 may be a potential therapeutic target for reversing primary platinum resistance in OC.

**Table 6:** Clinical data of 30 HGSC patients

Stratified by platinum reaction			
	Resistance	Sensitivity	*p* Test
n	10	20	
Age (mean (SD))	54.80 (7.41)	58.45 (7.04)	0.199
Stage (%)			0.639
I	0 (0.0)	1 (5.0)	
II	0 (0.0)	2 (10.0)	
III	8 (80.0)	14 (70.0)	
IV	2 (20.0)	3 (15.0)	
CD8 (mean (SD))	3.80 (1.75)	8.75 (3.51)	< 0.01
PDPN (mean (SD))	8.90 (3.18)	4.35 (2.13)	< 0.01
LRRC15 (mean (SD))	9.70 (2.36)	4.85 (2.43)	< 0.01

## Discussion

Ovarian cancer (OC) is the most common malignancy of the female reproductive system, especially in developing countries, with a five-year survival rate of less than 45%, and its incidence is still on an upwards trend. The ovaries are secluded in the pelvis, and early lesions have nonspecific symptoms, so most patients with ovarian cancer are diagnosed at a late stage, and the prognosis is poor, especially for high-grade serous ovarian cancer (HGSC). The current first-line treatments of ovarian cancer are paclitaxel or doxorubicin liposome combined with platinum-based chemotherapy [47]. In recent years, the research progress of targeted therapy has shown that PARP inhibitors are effective for platinum-sensitive ovarian cancer, and the 5-year survival rate has been significantly improved; however, primary platinum resistance is still the bottleneck of drug therapy for ovarian cancer [48]. Targeted immunotherapy has greatly improved the prognosis of cancer patients, and programmed death receptor 1 (PD-1)/programmed death ligand 1 (PD-L1) antibodies can induce a therapeutic effect via a strong and long-lasting reaction. However, these reactions occur in only a portion of patients [49].

In the process of tumour development and considering the importance of self-evident tumour cells, an increasing number of scholars have come to realize that tumour stromal cells are an important "accomplice" of the interaction between tumour cells and are closely involved in the regulation of tumour biological behaviour. Different solid tumours have different proportions of parenchyma and stroma, but in general, the stromal components of solid tumours account for more than 50% of the total tumour. Divided by the molecular phenotype, the mesenchymal subtype of ovarian cancer has the worst biological behaviour and shows a dense, abundant stroma [3]. In recent years, with the development of targeted therapy, tumour stromal targeted therapy has gradually been focused on and compared to the characteristics of the tumour parenchyma cells, which are prone to acquiring mutations, and the relative stability of the interstitial cells of many tumours [50–52]. Solid tumour interstitial fibroblasts, macrophages, endothelial cells, and a number of different extracellular matrix components support the structure of tumour growth. It positively regulates tumour growth, but the degree of these components may impair the host immune response and may contribute to the infiltration of immune cells.

The LRRC superfamily has a variety of functions, such as cell adhesion and signal transmission, extracellular matrix assembly, platelet aggregation, neuronal development, RNA treatment, pathogenic bacteria to host cell adhesion and intrusion, plant resistance and pathogenic identification. However, whether the LRRC superfamily plays a role in the tumour microenvironment, prognosis and response to treatment and its tumour immunology background are unknown, and there is a lack of scientific evidence. The bioinformatics analysis showed that the most enriched functional proteins of LRRC15 and LRRC32 were TGFB1 and TGFB2. This discovery suggests that the LRRC superfamily and TGF superfamily may have the same positive feedback regulation mechanism in the microenvironment of ovarian cancer and may jointly promote the invasion and metastasis of ovarian cancer.

A total of 515 OC samples (427 tumour samples + 88 normal samples) were downloaded from the TCGA and GTEx databases. Only LRRC15 was consistently highly expressed in solid tumours of ovarian cancer and was related to pathological stage, original therapy outcome and overall survival (P < 0.05). The Cox proportional hazards model and nomogram model showed that high LRRC15 expression was more likely to lead to adverse outcomes (HR = 1.76, *p* < 0.05). And we will conduct cross and external validation to improve the accuracy and credibility of the model in follow-up research. LRRC15 has recently been confirmed to be overexpressed in many solid tumours. LRRC15 is expressed in many solid tumours and interstitial fibroblasts and is expressed on cancer cells directly from a set of mesenchymal origins (e.g., sarcoma, melanoma, and glioblastoma tumours) [53–55]. A previous study found that compared with matched primary tumours, LRRC15 was highly expressed in intestinal OC metastasis samples and was an active promoter of omentum metastasis [56]. Furthermore, in immunotherapy clinical trials involving more than 600 patients with six kinds of cancer, LRRC15 + CAF displayed signal levels with anti-PD-L1, indicating a poorer response to treatment. Research on targeting the tumour microenvironment to enhance nonimmunological factors in cancer patients to improve the immune checkpoint blockade response is important [57]. Moreover, the first-in-human phase I study of ABBV-085, an antibody‒drug conjugate targeting LRRC15, was conducted [58]. This study observed preliminary antitumour activity of ABBV-085 in osteosarcoma and UPS patients. However, the significance of LRRC15 as a stromal marker in the immune infiltration and primary platinum resistance of ovarian cancer has not been confirmed. Therefore, we also evaluated the relationships between LRRC15 and immune cell infiltration and CAF infiltration in HGSC through TIMER2.0. The results showed that the high expression of LRRC15 contributed to the invasion of CAFs, which may promote the development of mesenchymal ovarian cancer. This also led to worse immune cell infiltration, indicating that LRRC15 may be used to screen people who would have a poor response to immunotherapy.

Our group first demonstrated high expression of LRRC15 in the MRC-5 cell line induced by TGF-β with qPCR. IHC then showed that LRRC15 is expressed in the stroma in most HGSC tissues. The diagnostic ROC curve showed that the predictive ability of LRRC15 was relatively accurate (AUC = 0.920, CI = 0.825–1.000) in predicting platinum resistance and sensitivity outcomes. The above observations highlight that LRRC15 is a representative biomarker of ovarian cancer stroma, which is related to the clinical prognosis and might be able to predict the primary platinum resistance of HGSC.

## Conclusions

Our research indicated that the LRRC superfamily may promote the invasion and metastasis of ovarian cancer via TGF-β signalling. LRRC15 plays a critical role in immune escape and CAF infiltration in HGSC. The LRRC15 signature can be used to predict the clinical prognosis and primary platinum resistance of high-grade serous ovarian cancer patients. Targeting LRRC15 might be beneficial in HGSC therapeutic management.

## Supplementary Information

**Additional file 1**: **Table S1** Independent sample T test results of PDPN and LRRC15 mRNA expression in TGF-beta treated and untreated MRC5 cell.

## Data Availability

The datasets presented in this study can be found in online repositories. The names of the repository/repositories and accession number(s) can be found in the article. The datasets generated and/or analysed during the current study are available from the corresponding author on reasonable request.

## Abbreviations

LRR: Leucine rich repeat sequence
LRRC: Leucine rich repeat containing protein
LRRC15: Leucine rich repeat containing protein 15
OC: Ovarian cancer
HGSC: High grade serous ovarian cancer
CAFs: Cancer-associated fibroblasts
PPI: Protein‒protein interaction
DEGs: Differentially expressed genes
IHC: Immunohistochemistry
PDPN: Podoplanin
EMT: Epithelial–mesenchymal transition
GSEA: Gene set enrichment analysis
EPIC: Estimation of the proportions of immune and cancer cells
