# Supplementary material for: LRRC superfamily expression in stromal cells predicts the clinical prognosis and platinum resistance of ovarian cancer
Source: BMC Med Genomics. 2023 Jan 18;16:10. doi: 10.1186/s12920-023-01435-9 (PMC9850808; doi:10.1186/s12920-023-01435-9)
Supplement: Supplementary file 1 — Additional file 1: Table S1 Independent sample T test results of PDPN and LRRC15 mRNA expression in TGF-beta treated and untreated MRC5 cell. [file 12920_2023_1435_MOESM1_ESM.docx]

Table S1 | Independent sample T test results of PDPN and LRRC15 mRNA expression in TGF-beta treated and untreated MRC5 cell.

Group   Group I [number] Group j [number] Statistics (t) degrees of freedom (df) statistics difference (J - I) Confidence interval (95%CI) P value

PDPN MRC5 [3] MRC5+TGF-β [3] 14.424 4 5.584 4.509 - 6.659 0.000

LRRC15 MRC5 [3] MRC5+TGF-β [3] 6.157 4 4.547 2.496 - 6.597 0.004

Independent sample T test results show that in the PDPN group, MRC5+TGF -β is higher than the average level of MRC5, and the difference between the two groups is 5.584 (4.509-6.659). The difference is statistically significant (T = 14.424, P <0.0011); In the LRRC15 group, the MRC5+TGF -β is higher than the average level of MRC5, and the difference between the two groups is 4.547 (2.496-6.597). The difference is statistically significant (T = 6.157, P = 0.004).
